# Diagnostic value of Xpert MTB/RIF assay on pleural tissue obtained via closed pleural biopsy among persons with presumptive tuberculous pleuritis

**DOI:** 10.7196/AJTCCM.2021.v27i1.120

**Published:** 2021-03-09

**Authors:** S D Yoo, G Abebe, J E Yoo, D Regassa, M Bezabih

**Affiliations:** 1 Department of Internal Medicine, Jimma University Institute of Health, Jimma University, Jimma, Ethiopia; 2 Department of Microbiology, Jimma University Institute of Health, Jimma University, Jimma, Ethiopia; 3 Department of Environmental Health, Jimma University Institute of Health, Jimma University, Jimma, Ethiopia; 4 Department of Epidemiology, Jimma University Institute of Health, Jimma University, Jimma, Ethiopia; 5 Department of Pathology, Jimma University Institute of Health, Jimma University, Jimma, Ethiopia

**Keywords:** biopsy, needle, diagnosis, pleural diseases, polymerase chain reaction, tuberculosis, pleural

## Abstract

**Background:**

Tuberculous pleuritis (TBP) is a common extrapulmonary tuberculosis that contributes to the tuberculosis burden. Xpert MTB/RIF assay is a promising method for rapid diagnosis of TBP. The diagnostic value of Xpert MTB/RIF assay in pleural tissue obtained via closed pleural biopsy among sputum acid-fast bacilli (AFB) smear-negative persons is not well studied.

**Objectives:**

To evaluate the diagnostic value of Xpert MTB/RIF assay on diagnosis of TB in pleural tissue obtained via blind closed pleural biopsy.

**Methods:**

Closed pleural biopsy using Cope needle was performed on adult patients who presented with lymphocyte predominant exudative pleural effusion. Xpert MTB/RIF assay was performed in parallel to pathology and mycobacterial culture of the pleural tissue specimen to determine its sensitivity and specificity. Final clinical diagnosis of TBP was determined by improvement in 2-months follow-up of anti-tuberculous treatment.

**Results:**

A total of 33 patients were included in the study. The median (interquartile range (IQR)) age was 27 (25 - 42) years. The sensitivity and specificity of Xpert MTB/RIF assay was 30% and 100% compared with Mycobacterium tuberculosis culture as the gold standard, and 20% and 95.7% compared with histopathology as the gold standard.

**Conclusion:**

Xpert MTB/RIF assay in pleural tissue obtained by closed pleural biopsy did not increase diagnostic yield, but it shortens time for diagnosis compared with conventional methods.

## Background

The burden of tuberculosis remains high, especially in developing countries. Some of these countries are reliant on age-old tuberculosis diagnostic tests such as sputum-smear microscopy, solid culture and chest radiography. Xpert MTB/RIF has recently become widely available in some developing countries. The assay shows high diagnostic accuracy for pulmonary tuberculosis and is highly recommended by the World Health Organization (WHO).^[1]^ However, its diagnostic accuracy for extrapulmonary tuberculosis is highly heterogeneous, yielding results ranging from 25.0% to 96.7%.^[2,3]^

Tuberculous pleuritis (TBP) is a common form of extrapulmonary tuberculosis. The diagnosis of tuberculous pleural effusion may be definitively established via demonstration of *Mycobacterium tuberculosis* in the pleural fluid or a pleural biopsy specimen. Measurement of adenosine deaminase (ADA) and interferon-gamma assay in the pleural fluid has also gained wide acceptance in the diagnosis of TB pleural effusions in developed countries.^[4]^ However, these tests are not readily available in most developing countries, including Ethiopia. Xpert MTB/RIF assay in pleural fluid has excellent specificity and lower sensitivity for TBP diagnosis.^[5,6]^ It is not considered as an initial evaluation test among patients with suspected TBP in a high-HIV/TB setting.^[7]^ Pathological diagnosis of TBP was confirmed via closed pleural biopsy, which is time-consuming. Meanwhile, the diagnostic value of the Xpert MTB/RIF assay in tissue biopsies has been reported to be high in some studies and the assay with pleural biopsy is regarded as a reliable tool for the diagnosis of TBP.^[8–13]^ However, the tissue specimens in these studies were obtained by a thoracoscopic biopsy that is not readily available in developing countries. Consequently, the diagnostic value of Xpert MTB/RIF assay in pleural tissue obtained by blind closed pleural biopsy that is readily available in developing countries has not yet been reported.

## Methods

### Participants

Adult patients who were consecutively admitted to the medical ward at Jimma University Medical Center in Jimma, Ethiopia, between September 2017 and December 2019 were screened for eligibility. Suspicion of TBP was defined by cough of at least 2 weeks with lymphocyte-predominant exudative pleural effusion. Patients with sputum that was smear-positive for acid-fast bacilli (AFB) or those who were already receiving anti-tuberculosis treatment at the time of initial evaluation were excluded from the study

### Data collection

Clinical and demographic information was collected by research officers from study participants using a standardised questionnaire after informed consent was obtained. Chest postero-anterior images were taken if they had not been taken before. Closed pleural biopsy was performed with Cope needle as per standard protocol by two physicians (SY and a resident supervised by SY). Biopsy specimens were divided into three pieces: one piece was sent to the Department of Pathology at Jimma University Medical Center for pathology staining and the other two pieces were sent to the Mycobacteriology Research Laboratory at Jimma University for *M. tuberculosis* culture using the Lowenstein-Jensen medium and Xpert MTB/RIF assay. Investigators followed the guidelines of Xpert MTB/RIF assay for pleural tissue analysis provided by WHO.^[14]^ Pleural fluid cytology was also performed to rule out the possibility of malignancy whenever the test was available. Vital status was assessed for all patients, either by telephone or in person 2 months after enrollment in the study. The final diagnosis of the participants was comprehensively evaluated based on the result of histology, positive *M. tuberculosis* culture from pleural tissue and clinical judgment after 2-months follow-up with anti-tuberculosis treatment.

### Statistical analysis

Bivariate analysis was performed to compare demographic and clinical characteristics, and outcomes between the patients with confirmed TBP and the non-TB patients, using the χ² test for dichotomous variables and the Mann-Whitney rank-sum test for continuous variables that are not normally distributed. Statistical analyses were performed using STATA version 14.0 (Stata Corp., USA) with the level of significance specified in reference to a two-tailed, type I error (p-value) of <0.05.

### Ethics

The Jimma University Ethical Review Board approved the study protocol before official commencement of the data collection (ref. no. IHPR3002). Informed consent was written in local languages (Amharic or Afaan Oromo).

## Results

**Fig. 1:**
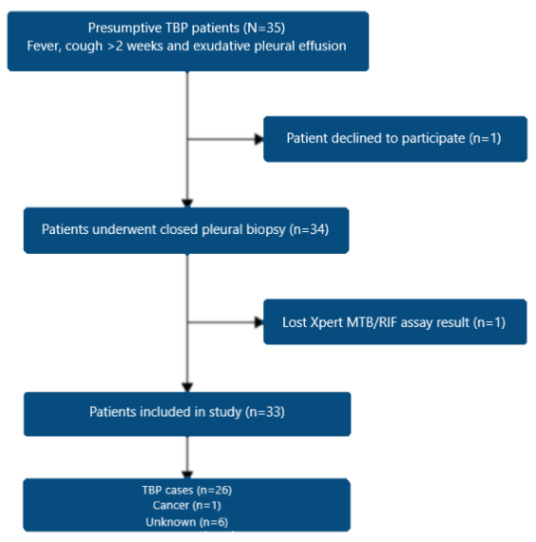
Number of eligible presumptive tuberculous pleuritis patients enrolled. (TBP = tuberculous pleuritis.)

A total of 35 adult patients were enrolled in the study but one patient declined to participate and another patient’s Xpert MTB/RIF assay results were lost (Fig. 1).

The median (interquartile range (IQR)) age of the study participants was 27 (25 - 42) years and the majority (55%) were female. There was no significant difference in the clinical findings between those who were diagnosed with TBP and those who had other diagnoses, except that the former were older and had more frequent chest pains than the latter (Table 1).

More than three-quarters of the study participants (78.8%; n=26/33) was diagnosed with TBP, 3% (n=1/33) was diagnosed with cancer and 18.2% (n=6/33) had no diagnosis. There were no clinical complications after pleural biopsy in all participants.

Less than a tenth (7.7%; n=2/26) of the patients with confirmed TBP were HIV-seropositive. Moreover, a majority of patients (53.8%; n=14/26) with confirmed TBP presented with right-sided pleural effusion, 38.5% (n=10/26) presented with left-sided pleural effusion and 7.7% (n=2/26) presented with bilateral pleural effusion (Table 2). There was no underlying parenchymal infiltration, cavitation or lymphadenopathy. The diagnosis of TBP was confirmed in 57.7% (n=15/26) of patients by comprehensive evaluation using histopathology, Xpert MTB/RIF assay and *M. tuberculosis* culture of pleural tissue. More than one-third (42.3%; n=11/26) of patients were clinically diagnosed. Three patients were Xpert MTB/RIF assay-positive and rifampicin sensitive. *M. tuberculosis* culture was positive in 10 patients including the three patients who were positive on Xpert MTB/RIF assay. Histopathology of 10 patients revealed typical TB with caseous necrosis. The percentage (95% confidence interval (CI)) sensitivity and specificity of Xpert MTB/RIF assay using pleural tissue obtained by closed pleural biopsy were 30% (18 - 45.2) and 100% (83.6 - 100) when compared with *M. tuberculosis* culture as the gold standard, and 20% (15.6 - 40.3) and 95.7% (78.3 - 99.2) when compared with histopathology as the gold standard (Table 3).

## Discussion

This is the first report on the diagnostic value of Xpert MTB/RIF assay on pleural tissue obtained by closed pleural biopsy in patients with AFB smear-negative sputum and presumptive TBP. A majority of TBP patients (57.7%; n=15/26) were confirmed by closed pleural biopsy using histopathology, Xpert MTB/RIF assay and *M. tuberculosis* culture. The majority of study participants (92.3%) were HIV seronegative. The diagnostic yield of pleural biopsy is known to be higher in HIV-uninfected patients (50 - 95%)^[15–17]^ than in HIV-infected patients (44 - 69%).^[18,19]^ The diagnostic yield of closed pleural biopsy was within the diagnostic range of both groups.

In our study, the sensitivity of the Xpert MTB/RIF assay on closed pleural biopsy tissue was 30% when compared with *M. tuberculosis* culture as the gold standard. We found the sensitivity to be lower than those reported in other studies. The reported sensitivity of PCR is highly heterogeneous, ranging between 45% and 100%, depending on the method of PCR assay, gold standards and method of biopsy.^[9-11,20-22]^ The conventional PCR studies^[20–22]^ reported higher sensitivity (56 - 100%) than the Xpert MTB/RIF assay studies (30 - 85%).^[9–11]^ Most studies obtained pleural tissue by either computed tomography-guided biopsy^[22]^ or thoracoscopic biopsy.^[10,11,20]^ Only one study obtained pleural tissue via closed pleural biopsy.^[21]^ The closed pleural biopsy study used conventional PCR and its sensitivity was 90%,^[21]^ which is much higher than our results. Further studies may be required to determine whether the differences in methods used to obtain the specimen affect the diagnostic yield of PCR assays. In our study, the sensitivity of Xpert MTB/RIF assay was higher when M. tuberculosis culture was the gold standard than when tissue pathology was the gold standard (30% v. 20%; p=0.05). This finding is consistent with other studies that used tissue pathology as the gold standard and reported a sensitivity of 45 - 52.2%.^[10,11]^ The studies that used *M. tuberculosis* culture as the gold standard reported sensitivity in the range of 85 - 100%.^[9,20,21]^ It is unclear why the sensitivity of the PCR assay is higher when using *M. tuberculosis* culture as the gold standard than when using tissue pathology as the gold standard. One possibility may be that the former might have used the same biopsy samples for both tissue culture and PCR assays while the latter may have used a different biopsy tissue for PCR assay and pathology, as we did in our study. The sensitivity of our study is slightly lower than the two studies that used Xpert MTB/RIF in thoracoscopic pleural biopsy specimens that showed sensitivity of 45% and 52%.^[10,11]^ It is known that the diagnostic sensitivity in pleural tissue obtained via closed percutaneous needle biopsy is lower than that obtained via thoracoscopy in TBP diagnosis.^[23,24]^

A tenth of patients (11.5%; n=3/26) with confirmed TBP were positive on Xpert MTB/ RIF assay in our study, which is a much lower detection rate compared with *M. tuberculosis* culture (38.5%) or histopathology (38.5%). Moreover, three patients with positive Xpert MTB/RIF results were also *M. tuberculosis* culture positive. This indicates that Xpert MTB/RIF assay did not contribute to diagnostic yield in our study. Nevertheless, Xpert MTB/RIF assay of pleural tissue is still useful because it provides rapid results and *M. tuberculosis* culture is not available in many parts of developing countries. Closed pleural biopsy should not be discouraged in such countries for several reasons:

(i) pleural tissue produces better diagnostic outcome than pleural fluid in TB diagnosis;^[10,20]^
(ii) tissue pathology may provide an opportunity to diagnose diseases other than TB; and
(iii) it has minimal complications in experienced hands although it is an invasive method.^[25]^

### Study limitation

The limitation of this study was that the statistical power was low because of the small sample size.

## Conclusion

Xpert MTB/RIF assay in pleural tissue obtained by closed pleural biopsy has low sensitivity and high specificity. It has a diagnostic value in developing countries where precise pleural fluid analysis is unavailable and considering its availability as well as rapid time to results.

## Figures and Tables

**Table 1:** Clinical findings of participants (N=33)

**Characteristics**	Overall, *n* (%)*	TBP, *n* (%)*	Others, *n* (%)*	***p*-value**
Age, median (IQR)	27 (25 - 42)	30 (23 - 45)	25 (23 - 40)	0.03
Gender (male)	15 (45.5)	9 (34.6)	6 (85.7)	0.09
HIV-seropositive	4 (12.1)	2 (7.7)	2 (28.6)	0.06
Lymphocytes, mean % in PE	65	76	60	0.07
Fever	25 (75.8)	23 (88.5)	2 (28.6)	0.12
Weight loss	24 (72.7)	20 (76.9)	4 (57.1)	0.63
DIB	27 (81.8)	24 (92.3)	3 (42.9)	0.05
Chest pain	21 (61.7)	20 (76.9)	1 (14.3)	0.01

TBP = tuberculous pleuritis

IQR = interquartile range

DIB = difficulty in breathing

PE = pleural effusion

*Unless otherwise specified

**TABLE 2:** Chest X-ray findings of patients with tuberculous pleuritis

**Characteristics**	***n* (%)**
Location of pleural effusion	
Right side	14 (53.8)
Left side	10 (38.5)
Both side	2 (7.7)
Amount of pleural effusion	
Small*	6 (23.08)
Moderate^†^	8 (30.77)
Massive^‡^	12 (46.15)

* Small = pleural effusion occupies <1/3 of unilateral lung fields.

^†^ Moderate = pleural effusion occupies >1/3 but <2/3 of the lung.

^‡^ Massive = pleural effusion occupies >2/3 of the lung field.

**Table 3:** Diagnostic value of Xpert MTB/RIF assay compared with different gold standards

**Parameters**	**TB culture,** **% (95% CI)**		**Histopathology,** **% (95% CI)**		**TB culture + histopathology,** **% (95% CI)**
Sensitivity	30 (18 - 45.2)		20 (15.6 - 40.3)		20 (12.1 - 33)
Specificity	100 (83.6 - 100)		95.7 (78.3 - 99.2)		100 (70.1 - 100)
PPV	100 (73.1 - 100)		66.7 (46.3 - 91.2)		100 (66.2 - 100)
NPV	23.3 (10.6 - 45.2)		26.7 (9.5 - 46.9)		40 (21.2 - 65.3)

TB = tuberculous pleuritis

CI = confidence interval

PPV = positive predictive value

NPV = negative predictive value
